# Typology and Triggers of Aggression in Organic Mental Disorders: Findings from a Prospective Study

**DOI:** 10.1192/j.eurpsy.2026.12007

**Published:** 2026-07-31

**Authors:** P. Kubíček, J. Vevera

**Affiliations:** 1 Department of Psychiatry, University Hospital; 2 Department of Psychiatry, Faculty of Medicine in Pilsen, Charles University, Pilsen; 3 Department of Psychiatry, Institute of Postgraduate Medical Education, Prague, Czech Republic

## Abstract

**Introduction:**

Aggressive behavior in psychiatric inpatients is a multidimensional construct encompassing impulsive, psychotic, and psychopathic domains, each with distinct neurobiological mechanisms and clinical implications. While most psychiatric patients are not violent, they remain at increased risk compared to the general population. In patients with organic mental disorders (ICD-10 F00–F09), aggression is rather challenging due to confusion, impaired insight and comorbidities. Systematic evaluation of aggressive incidents and their triggers is limited, despite its importance for developing targeted interventions.

**Objectives:**

This study aimed to pilot the systematic assessment of aggression in patients with organic mental disorders, classify incidents into impulsive, psychotic, and psychopathic domains, and analyze their immediate triggers, consequences, and management strategies.

**Methods:**

During an 8-month period, incidents were prospectively monitored in an acute adult psychiatric department in the Czech Republic. Only patients with organic mental disorders (ICD-10 F00–F09) were included. Aggressive behaviors were recorded using the Modified Overt Aggression Scale (MOAS). A violent incident was defined as a physically aggressive attack against people or property with a weighted score ≥3. Self-harm was analyzed separately, autoaggression was not included. Attacks were classified into impulsive, psychotic, or psychopathic domains using the Assault Interview Checklist. The Staff Observation Aggression Scale (SOAS) was used to capture context, targets, consequences, and management.

**Results:**

15 aggressive incidents from 12 patients were analyzed - 11 attacks (73%) were impulsive and 4 (27%) psychotic, no psychopathic incidents were identified. SOAS data revealed frequent triggers such as confusion, refusal of medication, hygiene-related care, and delirium. Aggression was mainly verbal, with physical acts involving hands or feet. Most incidents targeted staff, occasionally fellow patients. Consequences were minor and required no medical treatment. Management strategies consistently included verbal de-escalation and brief seclusion or gentle restraint. No severe injuries occurred.

**Conclusions:**

Impulsive aggression was the most common subtype among patients with organic mental disorders, followed by psychotic aggression, while psychopathic aggression was absent. Confusion, treatment refusal, and basic care activities were frequent situational triggers. Differentiating aggression domains is clinically relevant - impulsive aggression may benefit most from de-escalation techniques, while psychotic aggression requires targeted antipsychotic treatment.

**Disclosure of Interest:**

None Declared

